# Mechanistic Basis of Octreotide Therapy in Neonatal Postoperative Chylothorax: A Translational Case Report

**DOI:** 10.3390/children13101422

**Published:** 2026-10-16

**Authors:** Carmine Mattia, Roberta Leonardi, Sofia Surdo, Emanuele Liotta, Roberta Rocca, Fiorella Guadagni, Martino Ruggieri, Giulia Malaguarnera

**Affiliations:** 1 Neonatal Intensive Care Unit, AOU Policlinico G. Rodolico San Marco, 95123 Catania, Italy; lorettamattia@hotmail.com (C.M.); eliotta15@gmail.com (E.L.); rocca.roberta.3a@gmail.com (R.R.); 2 Postgraduate Training Programme in Pediatrics, Department of Clinical and Experimental Medicine, University of Catania, 95123 Catania, Italy; 3 Unit of Pediatric Clinic, Department of Clinical and Experimental Medicine, University of Catania, 95123 Catania, Italy; 4 Independent Researcher, 95100 Catania, Italy; s.surdo98@yahoo.com; 5 Department of Human Sciences and Quality of Life Promotion, San Raffaele Roma Open University, 00166 Rome, Italy; fiorella.guadagni@uniroma5.it (F.G.); giuliaannamaria.malaguarnera@uniroma5.it (G.M.)

**Keywords:** octreotide, somatostatin receptor, SSTR2, SSTR5, neonatal pharmacology, lymphatic contractility, congenital diaphragmatic hernia, postoperative chylothorax, translational pharmacology, developmental pharmacokinetics

## Abstract

Background and Clinical Significance: Octreotide is widely used off-label for neonatal postoperative chylothorax, although dosing is not standardised, and the supporting evidence comes mainly from case reports and case series. Somatostatin receptor (SSTR) subtypes 2 and 5 (SSTR2 and SSTR5) are expressed in the human thoracic duct, which provides a biological rationale for its use. Case Presentation: A term female neonate developed a massive left-sided chylothorax on initiation of enteral feeding, eight days after repair of a left congenital diaphragmatic hernia (CDH). Chest drainage yielded milky fluid with high triglyceride levels. Management comprised pleural drainage, total parenteral nutrition, fluid restriction, albumin, furosemide, antibiotics and continuous intravenous octreotide, started at 2 μg/kg/h and increased by 2 μg/kg/h daily to 12 μg/kg/h. Drainage declined after about 10 days, and radiological resolution after 20 days of treatment allowed for drain removal, extubation and reintroduction of feeding with a medium-chain triglyceride formula. Octreotide was tapered after 30 days. No treatment-related adverse events were observed. Conclusions: In this single case, postoperative chylothorax resolved during multimodal conservative management that included step-dose-escalated octreotide; the contribution of octreotide cannot be separated from that of the concurrent therapies. We discuss the differential diagnosis of pleural effusion after CDH repair and summarise the experimental rationale for octreotide, namely reduced splanchnic lymph production and an increased thoracic duct contraction rate ex vivo, which was not assessed in our patient. Prospective studies are needed to define evidence-based dosing and monitoring.

## 1. Introduction

Postoperative chylothorax is a severe complication of neonatal thoracic surgery, particularly following repair of congenital diaphragmatic hernia (CDH). Reported in approximately 7–28% of operated patients, it is associated with prolonged mechanical ventilation, nutritional depletion, immunological impairment, electrolyte disturbances, and extended hospitalization, thereby significantly increasing neonatal morbidity and healthcare burden [1,2,3,4]. The condition results from disruption of the thoracic duct or its tributaries during surgical manipulation, leading to continuous leakage of lymph into the pleural cavity.

Current management is based on conservative strategies aimed at reducing lymph production while maintaining adequate nutritional support. These include pleural drainage, total parenteral nutrition, and enteral feeding with medium-chain triglyceride (MCT)-based formulas. Although these measures successfully resolve many cases, persistent chyle leakage often requires additional pharmacological treatment before surgical interventions such as thoracic duct ligation or pleurodesis are considered [2,5].

Among the available pharmacological options, octreotide—a long-acting synthetic analogue of somatostatin—has become the most widely used off-label agent for neonatal chylothorax. Nevertheless, its clinical use remains largely empirical because no standardized dosing protocols have been established and current recommendations rely almost exclusively on case reports, case series, and retrospective observational studies [6,7,8]. Consequently, considerable variability exists regarding the optimal timing of treatment initiation, dose escalation, treatment duration, and monitoring strategies.

For many years, the beneficial effects of octreotide were primarily attributed to indirect physiological actions, including inhibition of gastrointestinal secretions, reduced intestinal fat absorption, and decreased splanchnic blood flow, all of which contribute to lowering lymph production. More recently, however, advances in lymphatic biology have substantially refined this view. Histological studies have demonstrated that somatostatin receptor (SSTR) subtypes 2 and 5 (SSTR2 and SSTR5), the principal pharmacological targets of octreotide, are expressed in smooth muscle cells of the human thoracic duct [9,10]. Furthermore, recent translational investigations have shown that octreotide increases the contraction rate of isolated human thoracic duct segments, suggesting that the drug may not only reduce chyle formation but also improve lymphatic transport under conditions of increased afterload [11].

These discoveries provide a mechanistic framework that links receptor pharmacology, intracellular Gi/o protein signalling, cyclic adenosine monophosphate (cAMP) inhibition, and lymphatic smooth muscle function to the clinical resolution of chylothorax. At the same time, neonatal developmental pharmacology introduces additional complexity. Immaturity of hepatic metabolism, renal clearance, plasma protein binding, and body fluid distribution significantly influence octreotide pharmacokinetics, making careful dose titration particularly important during the neonatal period [12]. In this age group, octreotide remains an off-label drug supported by limited safety data [13].

In this context, individual clinical observations may offer valuable translational insights when interpreted in light of current molecular evidence. We therefore report the resolution of postoperative chylothorax in a term neonate following CDH repair during multimodal conservative management that included continuous intravenous octreotide administered according to a stepwise dose-escalation protocol. Rather than presenting the case as an isolated clinical observation, we integrate it with current evidence on octreotide receptor pharmacology, lymphatic physiology, and neonatal developmental pharmacology to provide a mechanistically oriented perspective on its therapeutic role in postoperative chylothorax.

## 2. Case Presentation

Following surgical repair of a left-sided congenital diaphragmatic hernia (CDH), a term neonate developed postoperative chylothorax that resolved during a multimodal conservative strategy including continuous intravenous octreotide.

The infant, a girl, was born at 38 weeks of gestation by elective caesarean section because of a previous maternal uterine scar, with a birth weight of 2955 g. Immediately after birth, severe respiratory distress required endotracheal intubation. Chest radiography confirmed the diagnosis of left-sided CDH, and the patient was transferred to our Neonatal Intensive Care Unit (NICU). After sedation and stabilization, surgical repair was performed 24 h after admission. Herniated abdominal viscera—including the stomach, small intestine, colon, and spleen—were reduced into the abdominal cavity, and the diaphragmatic defect was closed. The postoperative chest radiograph showed partial re-expansion of the left lung, suggestive of pulmonary hypoplasia, with a residual basal pneumothorax.

On postoperative day 8, shortly after enteral feeding had been introduced, the infant experienced abrupt respiratory deterioration characterized by oxygen desaturation (SaO_2_ 75%), pallor, cutaneous mottling, and increasing respiratory distress.

**Figure 1:**
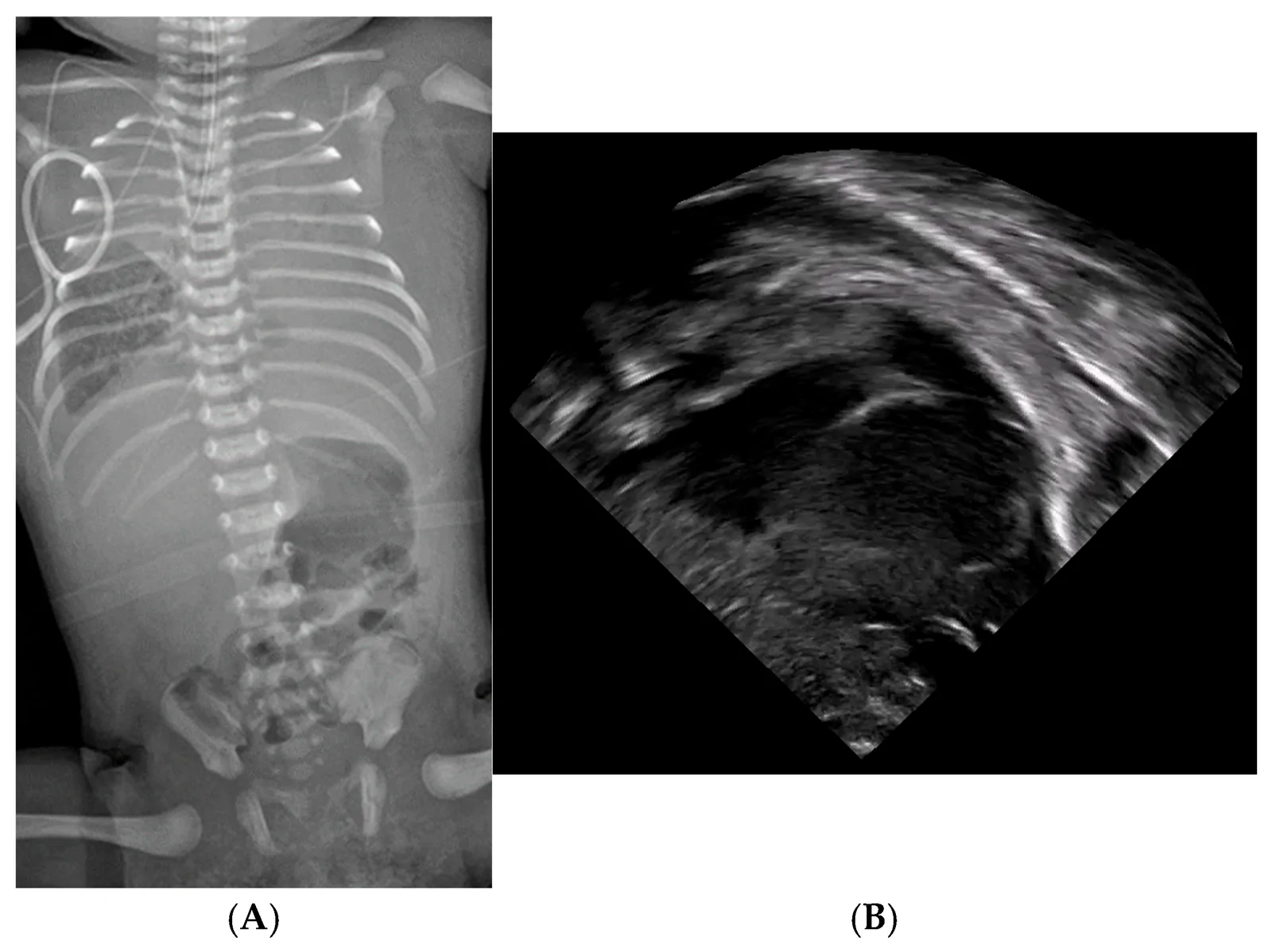
**Multimodal imaging assessment of postoperative chylothorax.** (**A**) Anteroposterior chest radiograph obtained on postoperative day 8 demonstrating complete opacification of the left hemithorax caused by a massive pleural effusion, with rightward mediastinal shift and compression of the residual aerated lung. (**B**) Bedside lung ultrasonography performed immediately after radiography confirming a large anechoic pleural effusion with near-complete collapse of the left lung, supporting urgent chest tube placement. The image is cropped to the scan sector.

A chest radiograph (Figure 1A) demonstrated complete opacification of the left hemithorax with rightward mediastinal displacement, findings highly suggestive of a large pleural effusion.

To further characterize the pleural collection, bedside lung ultrasonography (Figure 1B) was immediately performed and confirmed the presence of a large anechoic pleural effusion causing near-complete compression of the left lung, thereby supporting the indication for urgent pleural drainage.

Chest tube insertion resulted in the immediate evacuation of approximately 80 mL of milky pleural fluid, with rapid improvement in respiratory status. Pleural fluid analysis showed an alkaline pH, low protein content, and a low leukocyte count and lactate dehydrogenase (LDH) level, a profile consistent with a transudate, together with high triglyceride levels (Table 1). These findings, together with the milky appearance of the fluid on initiation of enteral feeding, confirmed the diagnosis of chylothorax; the accepted diagnostic criteria are a pleural fluid triglyceride concentration above 110 mg/dL and postoperative milky pleural drainage after enteral feeding [14,15].

A conservative therapeutic strategy was initiated, including pleural drainage, total parenteral nutrition, fluid restriction, intravenous albumin supplementation (20 mL/kg), continuous furosemide infusion (0.4 mg/kg/h), and broad-spectrum antibiotic therapy. Continuous intravenous octreotide was administered through a dedicated line at an initial dose of 2 μg/kg/h. The infusion rate was increased by 2 μg/kg/h every 24 h according to the clinical response until a maximum dose of 12 μg/kg/h was reached. Chylous output through the chest drain reached 30 mL/kg/day. Blood glucose, abdominal findings and feeding tolerance were monitored throughout the infusion.

Progressive reduction in pleural drainage became evident after approximately 10 days of treatment.

**Figure 2:**
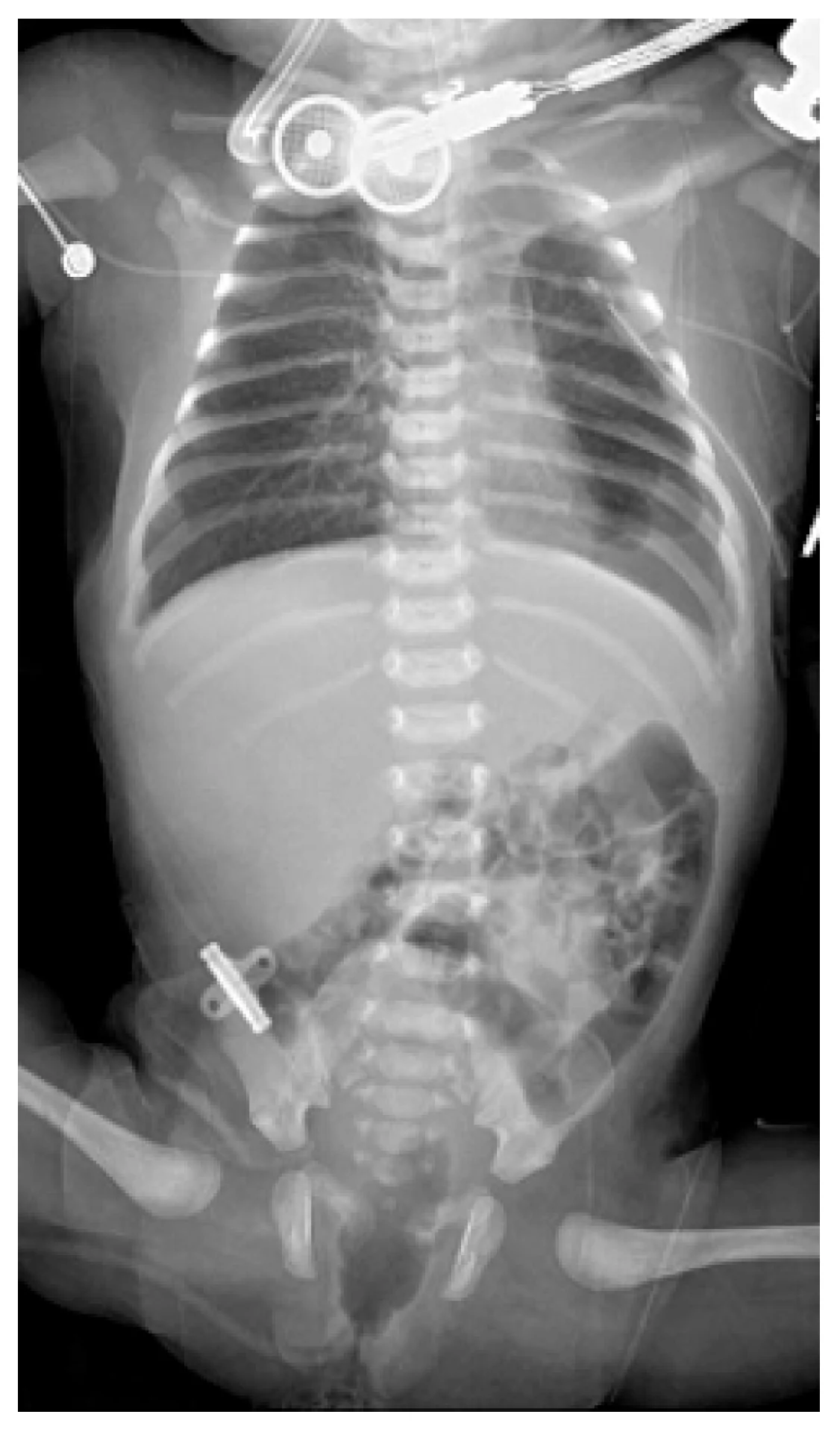
Anteroposterior chest radiograph after resolution of chylothorax (treatment day 20). Complete re-expansion of the left lung is demonstrated, with a well-positioned chest drain prior to its removal. The mediastinum has returned to its normal position.

A follow-up chest radiograph (Figure 2) obtained on treatment day 20 demonstrated complete resolution of the pleural effusion with full re-expansion of the left lung and restoration of normal mediastinal position. These findings allowed for chest tube removal, extubation, and gradual reintroduction of enteral feeding with a medium-chain triglyceride (MCT)-based formula while octreotide therapy was maintained.

**Figure 3:**
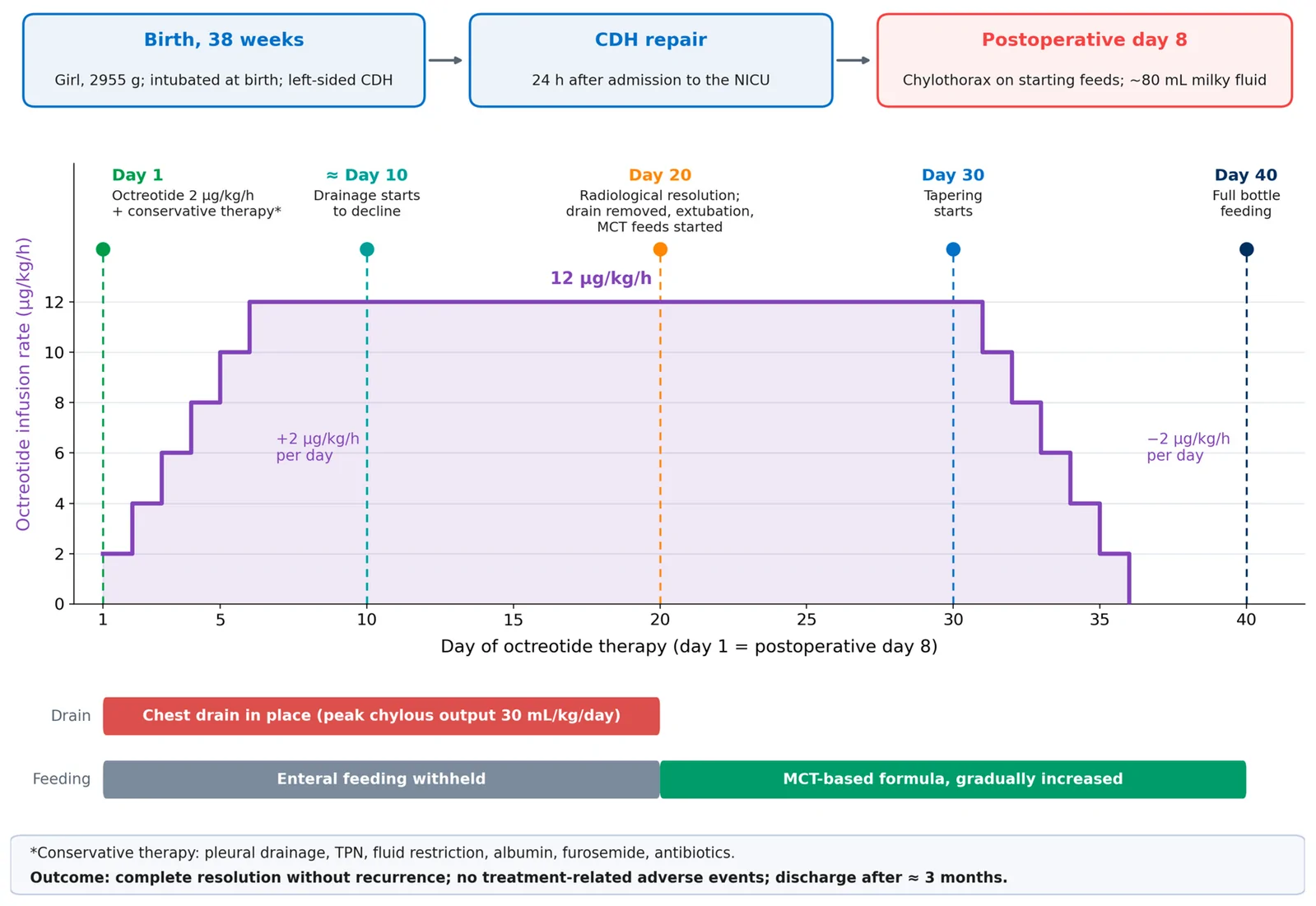
**Timeline of the clinical course and octreotide therapy.** The curve shows the octreotide infusion rate by day of therapy; the bars show the duration of chest drainage and the feeding regimen, and the markers indicate the main clinical events. The reduction in chyle leakage and the radiological resolution occurred while octreotide was being escalated together with pleural drainage, total parenteral nutrition, fluid restriction, albumin, furosemide and antibiotics; the individual contribution of octreotide cannot be separated from that of these concurrent therapies. Day 1 of therapy corresponds to postoperative day 8. CDH, congenital diaphragmatic hernia; MCT, medium-chain triglyceride; NICU, neonatal intensive care unit; TPN, total parenteral nutrition.

Octreotide was continued at 12 μg/kg/h until day 30 of treatment and was then tapered by 2 μg/kg/h per day until discontinuation. Throughout treatment, no drug-related adverse events—including hyperglycaemia, hypoglycaemia, gastrointestinal intolerance, and necrotising enterocolitis (NEC)—were observed. The infant achieved full bottle feeding by day 40, remained clinically stable without recurrence of pleural effusion, and was discharged after approximately three months of hospitalization. The clinical course is summarised in Figure 3.

## 3. Discussion

### 3.1. Clinical Course

Postoperative chylothorax remains one of the most challenging complications following congenital diaphragmatic hernia (CDH) repair. As illustrated in Figure 3, conservative management was complemented by gradual octreotide dose escalation. Chyle leakage decreased while pleural drainage, total parenteral nutrition, fluid restriction, albumin, diuretics and antibiotics were being given concurrently; the individual contribution of octreotide therefore cannot be disentangled from that of the other therapies, and causality cannot be inferred from a single case. The time course is nevertheless compatible with the mechanisms discussed below.

Persistent loss of chyle compromises respiratory function, nutritional status, immune competence, and fluid-electrolyte homeostasis, often prolonging intensive care and delaying recovery. Although conservative treatment remains the first-line approach, the optimal pharmacological management of refractory cases is still undefined because current evidence relies almost exclusively on observational studies, case series, and case reports [5,6,7,8,16,17].

In the present case, postoperative neonatal chylothorax resolved during multimodal conservative management that included a structured octreotide dose-escalation protocol, without further surgical intervention and without treatment-related adverse events.

### 3.2. Differential Diagnosis of the Postoperative Effusion

Pleural effusion is common after CDH repair and is not always chylous. In one series, 30% of infants required drainage of a postoperative effusion [18], whereas laboratory-confirmed chylothorax was reported in 18.6% of 396 neonates with CDH [19]. Several alternative causes were therefore considered in our patient. A serous effusion filling the space left by the hypoplastic lung, or a transudate related to fluid overload, hypoalbuminaemia or right heart failure [19], would be compatible with the low protein, cell and LDH content of the fluid but not with its milky appearance and high triglyceride level. Haemothorax was excluded by the appearance of the fluid, and empyema or a parapneumonic effusion was unlikely given the low leukocyte count and LDH level and the absence of infectious complications. Ultrasonography showed a large free pleural effusion (Figure 1B), which argued against early recurrence of the hernia as the cause of the opacified hemithorax. Finally, leakage of lipid-containing parenteral nutrition from a central venous catheter can produce a milky, triglyceride-rich effusion that mimics chylothorax and is distinguished by a high pleural fluid glucose content [20]. In our patient, the onset on initiation of enteral feeding, the prolonged course over about three weeks, and the location on the side of the repair, as in all cases of one CDH series [2], favoured a lymphatic leak over catheter-related extravasation, although this distinction rests on clinical grounds.

### 3.3. Mechanistic Rationale for Octreotide

The mechanisms summarised in this section are derived from published experimental and translational studies. None of them was measured in our patient, and the case neither supports nor refutes them; they are presented as the biological rationale for a treatment whose clinical efficacy remains unproven.

Octreotide is a synthetic cyclic octapeptide and a long-acting analogue of somatostatin with high affinity for somatostatin receptor subtype 2 (SSTR2) and subtype 5 (SSTR5), while exhibiting considerably lower affinity for SSTR1, SSTR3, and SSTR4 [10]. These receptors belong to the family of G-protein-coupled receptors (GPCRs) and are primarily coupled to inhibitory Gi/o proteins, which regulate multiple intracellular signalling pathways involved in secretion, vascular tone, and smooth muscle function.

Using immunohistochemical analysis, Jiang et al. demonstrated that both SSTR2 and SSTR5 are expressed in the smooth muscle cells of the human thoracic duct, providing direct anatomical evidence that the lymphatic system itself may be a pharmacological target for octreotide [9]. This finding extended the understanding of octreotide from a purely systemic antisecretory agent to a drug potentially capable of directly modulating lymphatic vessel function.

Binding of octreotide to SSTR2 and SSTR5 activates inhibitory Gi proteins, leading to suppression of adenylate cyclase activity and a consequent reduction in intracellular cyclic adenosine monophosphate (cAMP) concentrations [10].

The decrease in cAMP reduces protein kinase A activity, modifies calcium handling, promotes membrane hyperpolarization through activation of inward-rectifier potassium channels, and inhibits voltage-gated calcium channels. Collectively, these signalling events regulate smooth muscle excitability and contractile activity. Their net effect on lymphatic muscle cannot be inferred from canonical signalling alone: a fall in cAMP reduces protein kinase A-mediated relaxation and thus favours contraction, whereas membrane hyperpolarisation and inhibition of calcium entry, described mainly in neuroendocrine cells, would be expected to reduce excitability. The direction of the effect has therefore been established empirically, in isolated human thoracic duct, where octreotide increased the contraction rate [11].

Within the lymphatic system, these molecular events influence the behaviour of lymphangions, the functional contractile units of collecting lymphatic vessels. Coordinated contraction of lymphangions generates the intrinsic pumping activity responsible for propelling lymph through the thoracic duct.

Activation of SSTR2 and SSTR5 within the splanchnic circulation induces moderate vasoconstriction, reducing mesenteric blood flow and consequently decreasing intestinal lymph formation. Simultaneously, inhibition of gastrointestinal endocrine and exocrine secretion reduces intestinal fluid secretion and lipid absorption, further limiting chyle production [10,13,21].

Beyond these systemic effects, recent translational evidence has demonstrated a direct action on the thoracic duct itself. Holm-Weber and colleagues showed that octreotide increases the contraction frequency of isolated human thoracic duct segments in a concentration-dependent manner. In the accompanying randomised, double-blind, cross-over study in 16 healthy adults, octreotide infusion did not change spontaneous lymphatic contraction frequency or lymph pressure measured by near-infrared fluorescence imaging but almost doubled the isovolumetric pressure on plethysmography, which the authors interpreted as an increased lymphatic drainage capacity under high afterload [11].

**Figure 4:**
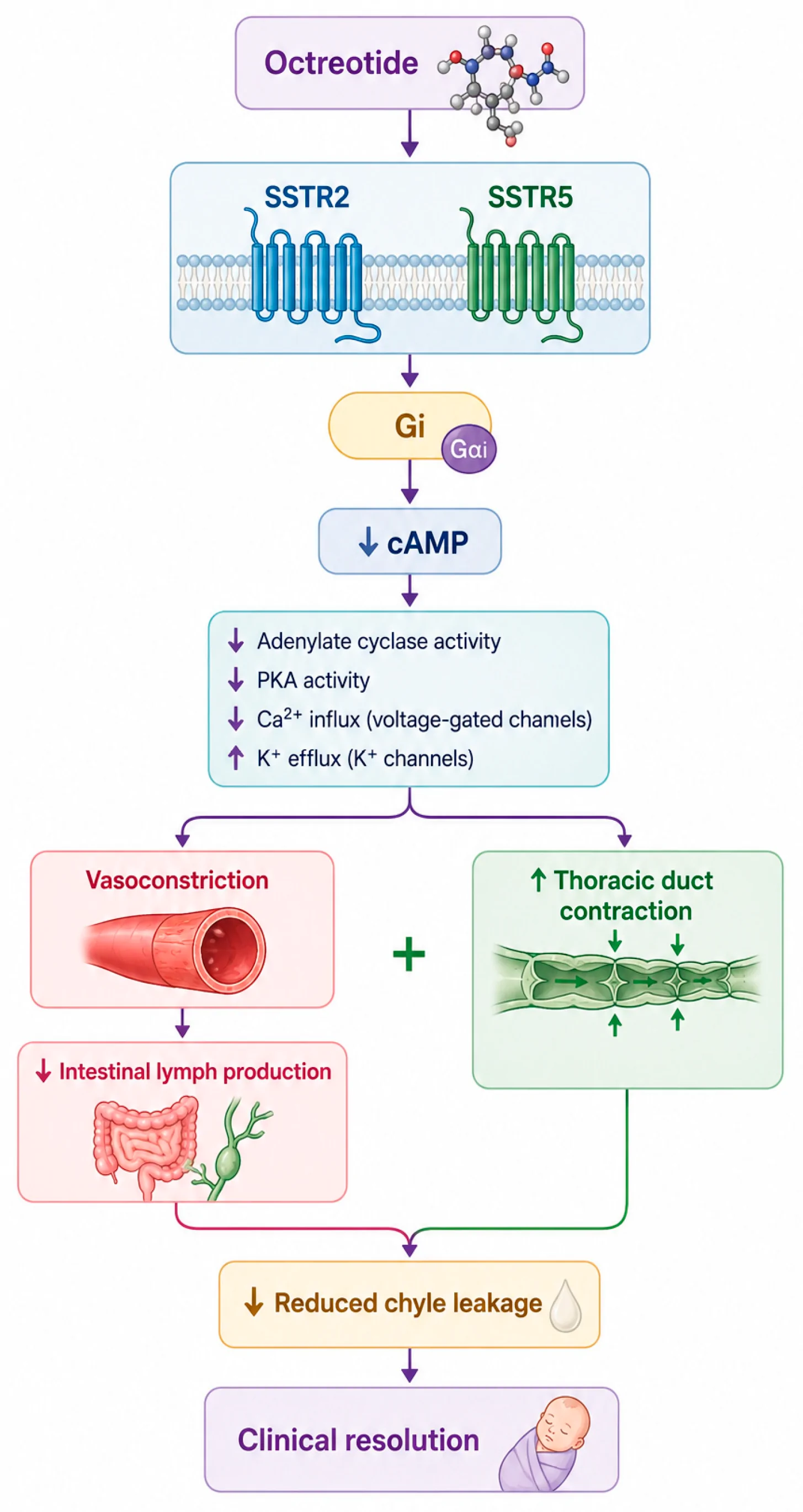
**Proposed molecular and physiological mechanisms of octreotide in neonatal postoperative chylothorax.** Activation of SSTR2 and SSTR5 by octreotide triggers Gi-protein-mediated inhibition of adenylate cyclase and intracellular cAMP signalling, leading to modulation of lymphatic smooth muscle function. In parallel, reduced splanchnic blood flow and decreased intestinal lymph production limit chyle formation, whereas enhanced thoracic duct contractility may improve lymphatic transport. Together, these complementary mechanisms may contribute to the reduction in chyle leakage. The scheme is based on published experimental data; none of these mechanisms was assessed in the present patient.

These observations are consistent with a dual mechanism of action, a reduction in chyle formation and enhancement in lymphatic drainage, although the latter has not yet been demonstrated in neonates or in patients with chylothorax (Figure 4, Table 2).

This dual mechanism may be particularly relevant after CDH repair, where surgical disruption and postoperative tissue remodelling increase lymphatic outflow resistance and impair physiological drainage [5,6,11].

### 3.4. Dosing, Pharmacokinetics and Generalisability

From a pharmacokinetic perspective, neonatal physiology differs substantially from that of adults. The larger extracellular water compartment, reduced plasma protein binding, immature hepatic metabolism, and incomplete renal clearance may significantly influence octreotide disposition, resulting in considerable interindividual variability [12]. These developmental characteristics support individualized dose titration rather than direct extrapolation of adult dosing regimens. In our patient, gradual escalation from 2 to 12 μg/kg/h allowed treatment to be tailored according to the clinical response, and no adverse events were observed.

The extent to which this mechanistic reasoning and dosing strategy can be generalised is uncertain. Our patient was a term neonate with a postoperative lymphatic leak. In preterm infants, the larger proportion of body water, lower plasma protein concentrations, lower glomerular filtration rate and less mature hepatic metabolism are expected to alter octreotide distribution and clearance further, so that exposure at a given weight-based infusion rate cannot be assumed to match that of a term infant [12]. To our knowledge, octreotide pharmacokinetics have not been characterised in preterm infants. The contractility data on which the proposed lymphatic mechanism rests were obtained in isolated human thoracic duct and in healthy adult volunteers [11], and, to our knowledge, neither receptor expression nor the contractile response has been studied in neonatal lymphatic tissue [9,11]; whether SSTR2 and SSTR5 are expressed and functional in the neonatal thoracic duct, and from which gestational age, is unknown. The immature intestinal circulation and the higher baseline risk of NEC in prematurity also argue for more cautious escalation and closer abdominal surveillance than in our patient. Congenital chylothorax differs from the postoperative form in that it often reflects abnormal lymphatic development rather than a focal leak from a structurally normal duct [6,22]; a reduction in lymph production may be relevant in both settings, whereas a benefit mediated by enhanced contractility presupposes functionally competent lymphatic smooth muscle. In the systematic review by Bellini et al., which included term and preterm newborns, octreotide was judged effective in 30 of 57 congenital and 9 of 27 acquired cases, a difference that was not statistically significant [6]. The dosing scheme described here should therefore not be extrapolated to preterm infants or to congenital chylothorax without dedicated pharmacokinetic and clinical data.

The current literature highlights substantial heterogeneity in octreotide administration, with considerable variation in starting doses, escalation schedules, treatment duration, and maximum infusion rates [6,7,8,22,23,24,25]. Nevertheless, despite this variability, most successful reports—including the present case—share a common therapeutic strategy consisting of continuous intravenous infusion with progressive dose adjustment according to clinical evolution. This observation suggests that individualized pharmacodynamic titration may be more relevant than adherence to a universal dosing protocol.

The dose used in our patient is also consistent with the available literature. Bellini et al. reported an overall response rate of approximately 47% among neonates treated with octreotide, with adverse events occurring in 14.3% of cases [6]. Although isolated reports have described successful treatment with infusion rates up to 20 μg/kg/h, no comparative data currently demonstrate that higher infusion rates are more effective, while higher doses may theoretically increase the risk of adverse effects [7,17]. In the present case, complete clinical and radiological resolution was observed during infusion at 12 μg/kg/h, given together with the other conservative measures, without hyperglycaemia, hypoglycaemia, gastrointestinal intolerance, or necrotising enterocolitis.

### 3.5. Safety and Monitoring

The safety of octreotide in neonates must be weighed against these uncertain benefits. In the largest available cohort (428 hospitalised infants, 490 courses), hypotension requiring vasopressors was the most frequent clinical adverse event (12%), NEC occurred in 2% of courses, and hyperglycaemia and hypoglycaemia occurred in 1 and 3 per 1000 infant-days, respectively [26]. In neonates treated specifically for chylothorax, side effects were reported in 12 of 84 patients (14.3%) [6]. Other effects described with octreotide include bradycardia, gastrointestinal disturbances such as abdominal distension and altered motility, cholelithiasis and hypothyroidism [8,13] (Table 3). NEC is the most serious concern and is biologically plausible, given that splanchnic vasoconstriction is part of the therapeutic action of the drug. Because the available reports are small and uncontrolled, adverse-event rates cannot be compared reliably across dose ranges: infusion rates of up to 20 μg/kg/h have been reported without major adverse events [7,17], but these data are too sparse to exclude dose-dependent harm. In our patient, 12 μg/kg/h was taken as the dose at which the response should be assessed, because it lies just above the range most commonly reported for continuous infusion (1–10 μg/kg/h) [6,7] and well below the 20 μg/kg/h reserved for refractory cases; further escalation would have been considered only if drainage had failed to decline. We suggest that dose escalation be accompanied by serial bedside blood glucose measurements, particularly after each dose change and during weaning, by continuous heart-rate and blood-pressure monitoring, and by daily assessment of abdominal distension, feeding tolerance and stool for blood, with enteral feeds advanced cautiously; liver and thyroid function tests are reasonable during prolonged courses.

### 3.6. Limitations and Future Directions

Several limitations should nevertheless be acknowledged. As a single-patient observation, this report cannot establish causality or define therapeutic efficacy. Clinical improvement probably resulted from the combined effects of pleural drainage, bowel rest with total parenteral nutrition, fluid restriction, albumin, diuretics and octreotide, and spontaneous sealing of the lymphatic leak over time cannot be excluded; the contribution of octreotide alone cannot be quantified. Published experience in CDH is itself conflicting: in one single-centre series, five of six infants treated with octreotide for chylothorax after CDH repair still required pleurectomy [2]. Finally, the pleural fluid had a transudate-like biochemical profile, whereas chylous effusions are usually lymphocyte-predominant exudates; transudative chylothorax is nevertheless well documented, accounting for 14% of cases in one series [27]. Moreover, pharmacokinetic measurements, receptor expression analyses, and circulating biomarkers were not available, preventing direct confirmation of the proposed molecular mechanisms in this individual patient.

Several measurements that are feasible in routine neonatal intensive care could test the proposed mechanism in individual patients. First, plasma octreotide concentrations can be measured by liquid chromatography–tandem mass spectrometry on small-volume or scavenged samples obtained at steady state after each dose step, allowing exposure to be related to drainage and compared with the concentrations that stimulate thoracic duct contraction ex vivo [11]. Second, pleural fluid is already available from the drain: daily output indexed to body weight, together with serial triglyceride concentration, total cell count and lymphocyte fraction, would distinguish a true fall in chyle flow from dilution. Third, the splanchnic effect can be followed non-invasively with Doppler flow velocities in the superior mesenteric artery or abdominal near-infrared spectroscopy before and after each dose change. Fourth, blood glucose and insulin, which are needed for safety monitoring, also provide a simple index of somatostatin-receptor engagement. Finally, lymphatic function can be imaged directly in specialised centres: near-infrared fluorescence lymphography has been used to quantify lymphatic contraction frequency and pressure in adults [11], and magnetic resonance techniques can characterise chylous effusions [28]. Collected prospectively and uniformly, for example within a multicentre registry, such data would allow exposure–response relationships to be described even where randomised trials are not feasible.

Future studies should move beyond descriptive clinical outcomes toward mechanism-oriented investigations integrating developmental pharmacokinetics, pharmacodynamics, receptor biology, and biomarkers of lymphatic function. Multicentre prospective registries and, where feasible, randomized controlled trials remain essential to establish evidence-based dosing strategies and identify predictors of therapeutic response in neonatal postoperative chylothorax [6,16]. Collectively, the available molecular, translational, and clinical evidence suggests that octreotide may act as a targeted pharmacological modulator of lymphatic physiology rather than as a purely empirical adjunctive therapy. The present case is compatible with this evolving mechanistic paradigm but does not test it.

## 4. Conclusions

In this single case, continuous intravenous octreotide was a feasible and well-tolerated component of the multimodal conservative management of postoperative chylothorax following congenital diaphragmatic hernia repair. Although its efficacy cannot be established from one patient receiving concurrent therapies, current experimental and translational evidence provides a plausible molecular rationale for its therapeutic application. SSTR2 and SSTR5 are expressed in human thoracic duct smooth muscle, and octreotide reduces splanchnic lymph production and increases the contraction rate of isolated thoracic duct; these mechanisms offer a biologically plausible, but unproven, explanation for the reduction in chyle leakage reported in clinical practice.

The mechanistic rationale summarised here (Table 2) is drawn from published experimental work and was not tested in this patient; it offers a framework for, not evidence of, a mechanism-based use of octreotide in neonatal chylothorax. Nevertheless, current evidence remains largely derived from observational studies and isolated clinical reports. Prospective multicentre studies integrating pharmacokinetics, pharmacodynamics, biomarkers of lymphatic function, and standardized treatment protocols are required to translate current empirical practice into evidence-based therapeutic recommendations. Ultimately, integrating molecular pharmacology with carefully documented clinical observations may accelerate the development of precision-based therapeutic strategies for rare neonatal disorders in which conventional randomized trials remain difficult to perform.

## Figures and Tables

**Table 1:** Pleural fluid analysis in the present case, reported relative to standard thresholds, compared with the typical profile of an exudate.

Parameter	Exudate	Our Patient (Chylothorax)
**pH**	Acid	Alkaline
**Total proteins**	>30 g/L	<10 g/L
**Colour**	Serous/cloudy	Milky
**LDH**	>200 U/L	<200 U/L
**Leukocytes**	>1000/μL	<1000/μL
**Cytology**	Neutrophilic leukocytosis	Scant mesothelium
**Triglycerides**	<110 mg/dL	High (diagnostic threshold: >110 mg/dL)

**Table 2:** Molecular basis of octreotide therapy in neonatal postoperative chylothorax.

Molecular Target	Molecular Mechanism	Experimental Evidence	Clinical Implication
SSTR2 and SSTR5	High-affinity binding of octreotide to SSTR2 and SSTR5 on thoracic duct smooth muscle cells	Immunohistochemistry of the human thoracic duct (Jiang et al. [9])	The thoracic duct is a potential direct pharmacological target of octreotide
Gi/o proteins	Inhibition of adenylate cyclase and reduction in intracellular cAMP	Receptor pharmacology review (Günther et al. [10])	Links receptor activation to intracellular signalling in smooth muscle
cAMP signalling	Modulation of calcium handling and membrane excitability	Experimental GPCR studies [10]	May modulate lymphatic smooth muscle contractility
Thoracic duct (lymphangions)	Concentration-dependent increase in contraction rate ex vivo; increased isovolumetric pressure in healthy adults	Human thoracic duct myography and randomised cross-over study (Holm-Weber et al. [11])	May improve lymph transport under high afterload and thereby reduce chyle leakage
Splanchnic circulation and gut	Splanchnic vasoconstriction with reduced gastrointestinal secretion and fat absorption	Clinical pharmacology studies and reviews [10,13]	Reduces intestinal lymph (chyle) production

**Table 3:** Octreotide-related adverse events reported in neonates and infants, and monitoring suggested during dose escalation.

Adverse Event	Reported Evidence	Suggested Monitoring
Hypotension	Vasopressor support required in 12% of a cohort of 428 hospitalised infants (490 courses) [26]	Continuous heart-rate and blood-pressure monitoring
Necrotising enterocolitis	2% of courses [26]; isolated cases in infants treated for chylothorax [8,16]	Daily abdominal examination, feeding tolerance and stool for blood; cautious advancement of enteral feeds
Hyperglycaemia and hypoglycaemia	1 and 3 per 1000 infant-days, respectively [26]; transient hyperglycaemia in case reports [8]	Bedside blood glucose after each dose change and during weaning
Gastrointestinal intolerance	Abdominal distension and feeding intolerance in case reports and reviews [8,16]	Abdominal girth, gastric residuals and stool pattern
Transient hypothyroidism	Reported in neonates treated for chylothorax [16]	Thyroid function tests during prolonged courses
Bradycardia, cholelithiasis	Listed among common adverse effects in the prescribing information [13]	Heart-rate monitoring; liver function tests and abdominal ultrasound if treatment is prolonged

## Data Availability

No new data were created or analyzed in this study. Data sharing is not applicable to this article.

## Abbreviations

The following abbreviations are used in this manuscript:

CDH	Congenital Diaphragmatic Hernia
cAMP	Cyclic Adenosine Monophosphate
Gi	Inhibitory G-protein
GPCR	G-Protein-Coupled Receptor
LDH	Lactate Dehydrogenase
MCT	Medium-Chain Triglycerides
NEC	Necrotising Enterocolitis
NICU	Neonatal Intensive Care Unit
SaO_2_	Arterial Oxygen Saturation
SSTR	Somatostatin Receptor
